# Bright Blue Light Emission of ZnCl_2_-Doped CsPbCl_1_Br_2_ Perovskite Nanocrystals with High Photoluminescence Quantum Yield

**DOI:** 10.3390/mi16080920

**Published:** 2025-08-09

**Authors:** Bo Feng, Youbin Fang, Jin Wang, Xi Yuan, Jihui Lang, Jian Cao, Jie Hua, Xiaotian Yang

**Affiliations:** 1 College of Information Technology, Jilin Engineering Research Center of Optoelectronic Materials and Devices, Jilin Normal University, Siping 136000, China; fengbosiping@126.com (B.F.); yuanx@jlnu.edu.cn (X.Y.); 2 Jilin Provincial Key Laboratory of Wide Bandgap Semiconductor Material Growth and Device Applications, Jilin Normal University, Changchun 130103, China; 3 College of Physics, Jilin Normal University, Changchun 130103, China; langjihui80@126.com (J.L.); caojianlxj@126.com (J.C.)

**Keywords:** ZnCl_2_-doping, CsPbCl_1_Br_2_, perovskite nanocrystals, variable-temperature photoluminescence spectroscopy, photoluminescence quantum yield

## Abstract

The future development of perovskite light-emitting diodes (LEDs) is significantly limited by the poor stability and low brightness of the pure-blue emission in the wavelength range of 460–470 nm. In this study, the Cl/Br element ratio in CsPbCl_x_Br_3−x_ perovskite nanocrystals (NCs) was modulated to precisely control their blue emission in the 428–512 nm spectral region. Then, the undoped CsPbCl_1_Br_2_ and the ZnCl_2_-doped CsPbCl_1_Br_2_ perovskite NCs were synthesized via the hot-injection method and investigated using variable-temperature photoluminescence (PL) spectroscopy. The PL emission peak of the ZnCl_2_-doped CsPbCl_1_Br_2_ perovskite NCs exhibits a blue shift from 475 nm to 460 nm with increasing ZnCl_2_ doping concentration. Additionally, the ZnCl_2_-doped CsPbCl_1_Br_2_ perovskite NCs show a high photoluminescence quantum yield (PLQY). The variable-temperature PL spectroscopy results show that the ZnCl_2_-doped CsPbCl_1_Br_2_ perovskite NCs have a larger exciton binding energy than the CsPbCl_1_Br_2_ perovskite NCs, which is indicative of a potentially higher PL intensity. To assess the stability of the perovskite NCs, high-temperature experiments and ultraviolet-irradiation experiments were conducted. The results indicate that zinc doping is beneficial for improving the stability of the perovskite NCs. The ZnCl_2_-doped CsPbCl_1_Br_2_ perovskite NCs were post-treated using didodecylammonium bromide, and after the post-treatment, the PLQY increased to 83%. This is a high PLQY value for perovskite NC-LEDs in the blue spectral range, and it satisfies the requirements of practical display applications. This work thus provides a simple preparation method for pure blue light-emitting materials.

## 1. Introduction

Perovskite nanocrystals (NCs) have attracted intense research attention because of their superior optoelectronic characteristics, which position them as attractive candidates for diverse applications including lighting, displays, photovoltaics, sensing, and lasing [1,2,3,4,5,6,7,8]. All-inorganic CsPbX_3_ (X = Cl, Br, I) perovskite NCs are inexpensive and exhibit excellent photophysical properties, including strong excitonic absorption, tunable luminescence, high photoluminescence quantum yield (PLQY), and narrow excitonic emission peaks; these properties are achieved by changing the material composition [9,10,11]. Over the past years, rapid progress has been achieved for green- and red-perovskite NC light-emitting diodes, but the emission efficiency and stability of blue-emitting NCs are significantly lower than those of perovskites emitting other colors, which substantially restricts their practical application [12,13]. Zhao and co-workers reported that by precisely controlling the synthesis conditions, CsPbBr_3_ nanorods with blue luminescence could be successfully prepared. By optimizing the ratio of ethyl acetate to isopropanol, the nanorods were purified, and the excess ligands were removed, endowing them with good stability [14]. In addition, Wu and co-workers reported the synthesis of 4-dodecylbenzenesulfonic acid-capped CsPbBr_3_ nanosheets without using any amines and elucidated their nucleation and growth mechanisms. The thickness of the amine-free nanosheets was controlled with single-layer precision, the emission wavelength could be adjusted between 437 and 504 nm, the photoluminescence quantum yield was as high as 80%, and the carrier relaxation was found to be slow [15]. The blue emission of perovskite emitters in the range of 450–490 nm can be modulated by changing their composition (mixed halide perovskites) [16,17]. In addition, Br^−^ and Cl^−^ vacancies can be generated due to their low formation energy. The reason for the low performance of blue-emitting perovskite NC-based LEDs compared with that of green- or red-emitting perovskite LEDs is the complete suppression of Cl^−^ vacancies in perovskite NCs. Both avoiding the loss of charge carriers and preventing halide migration are required. For example, the first developed pure-blue perovskite LED, which utilized CsPbBr_x_Cl_3−x_ NCs with a mixed bromide/chloride (Br/Cl) composition, was fabricated via the hot injection method and exhibited an external quantum efficiency (EQE) of 0.07% at 455 nm [18]. Furthermore, Tepliakov and co-workers studied the near-infrared photoluminescence of Yb^3+^-doped hybrid halide CsPbCl_x_Br_3−x_ NCs as a function of temperature, and found a strong dependence of the host perovskite matrix on the stoichiometry. They reported that trap-mediated energy transfer was the main photo sensitization mechanism in Yb^3+^-doped CsPbCl_x_Br_3−x_ NCs [19]. Therefore, developing pure-blue perovskite LEDs based on CsPbCl_x_Br_3−x_ NCs with both good stability and excellent optoelectronic performance remains a considerable challenge.

CsPbBr_3_ is a typical all-inorganic halide perovskite, that exhibits an efficient, bright, monochromatic, narrow emission and has thus attracted widespread attention [20,21]. The PLQY is an important factor in determining the performance of all-inorganic halide perovskite NC-based devices. Defects and traps in NCs can act as nonradiative recombination centers, reducing the PLQY of these devices. For example, Liu and co-workers synthesized CsPbBr_3_ nanosheets with five (PbBr_6_)^4−^ single layers and comprehensively studied them through femtosecond transient absorption spectroscopic, steady-state absorption, and photoluminescence measurements. They determined the biexciton Auger recombination time and the trapped exciton lifetime. Additionally, the origin of the low-energy tail emission in the obtained photoluminescence spectra was also investigated [22]. Varnakavi and co-workers proposed a method for the in situ synthesis of blue light-emitting two-dimensional CsPbBr_3_ nanosheets rich in Br. ZnBr_2_ was used as the Br precursor to enhance the adsorption capacity of Br ions. This effectively passivated the surface defects, improved the PLQY, enhanced the stability of the nanosheets, and made them suitable for display and lighting applications [23]. Through meticulous surface engineering techniques, Huang and co-workers effectively harnessed the robustly bonded N-dodecylbenzenesulfonic acid to confer the CsPbBr_3_ nanosheets an atomic crystal-like degree of structural rigidity. By optimizing the intermediate reaction phase, a monolayer Cs_x_O shell was successfully formed, which completely encapsulated the surface of the nanosheets. This accomplishment led to the realization of a highly stable and rigid crystal structure within the nanosheets, along with a uniformly ordered arrangement on their surface. Consequently, the resultant CsPbBr_3_ nanosheets demonstrated intense blue photoluminescence, and the electroluminescence bandwidth reached a groundbreaking value of 15 nm, setting a new record in this field [24].

As is well known, doping can endow the host semiconductor materials, especially perovskite NCs, with exciting and novel properties. First, Chen and co-workers reported that the luminescence and thermal stability of CsPbBr_3_ quantum dot glasses could be enhanced through doping with Eu^3+^ ions. The research showed that the introduction of Eu^3+^ ions changed the electronic structure of the quantum dots and optimized the energy transfer process, thus significantly improving the luminescence efficiency. At the same time, thermal stability experiments revealed that the performance of the doped quantum dot glasses in high-temperature environments had been greatly enhanced, laying a foundation for their application in optoelectronic devices under high-temperature working conditions [25]. Second, Wang and co-workers reported that CsPbBr_3_ quantum dots doped with In^3+^ ions exhibited stronger emission and better stability compared with the original, undoped quantum dots. The doped CsPbBr_3_ quantum dots had been used as green emitters for manufacturing white light-emitting diodes (LEDs), and the color gamut reached 127% of the standard set by the National Television Standards Committee (NTSC) [26]. Third, Jiang and co-workers reported that using the vapor deposition method to incorporate trace amounts of KBr into the perovskite, the quality of the thin film was effectively improved, the concentration of surface defects was reduced, and the transport and extraction of carriers were enhanced. The prepared perovskite solar cells had an average visible-light transmittance of 44%, an open-circuit voltage as high as 1.55 V, and a power conversion efficiency of 7.28% [27]. Based on existing studies on the doping of perovskite NCs, using smaller-radius ions to partially replace Pb^2+^ is considered to be a good strategy to stabilize the phase of CsPbI_3_ NCs and enhance the PLQY of the doped perovskite NCs. In particular, the introduction of Zn^2+^ improves the PLQY and phase stability of CsPbI_3_ [28]. It should be noted that the Zn^2+^ cation is an effective dopant due to its special physicochemical properties [29]. Therefore, incorporating Zn^2+^ ions into the perovskite structure is an effective strategy to enhance the optical properties of CsPbBr_3_ perovskite NCs. The PLQY of CsPbCl_1_Br_2_ NCs has been investigated, but no consensus has been reached regarding the PLQY of ZnCl_2_-doped CsPbCl_1_Br_2_ NCs subjected to post-treatment. We simultaneously tune the content of zinc and chlorine in ZnCl_2_-doped CsPbCl_1_Br_2_ perovskite NCs, adjust the position of the emission peak, and study the influence of zinc and chlorine on the luminescence properties (and especially the PLQY) of CsPbCl_x_Br_3−x_ NCs. To the best of our knowledge, no report has studied the PL of ZnCl_2_-doped CsPbCl_1_Br_2_ NCs as a function of temperature.

In this study, ZnCl_2_-doped CsPbCl_1_Br_2_ perovskite NCs were prepared via a facile hot injection method to realize blue emission with the possibility of tuning the PL peak from 460 to 475 nm. An emission peak is observed at 460 nm with a full width at half maximum (FWHM) of 18 nm, and the PLQY can reach 73%. The structural properties and PL temperature dependence of CsPbCl_1_Br_2_ and ZnCl_2_-doped CsPbCl_1_Br_2_ were also investigated in detail. It was found that after the post-treatment, the PLQY increases to 83%. This study provides a relatively simple preparation method for the development of pure blue-emission perovskite NCs.

## 2. Experimental Section

### 2.1. Materials

Oleic acid, oleylamine, 1-octadecene, lead chloride, lead bromide, zinc chloride, cesium carbonate, tetramethyl ammonium bromide, ethyl acetate, and didodecylammonium bromide were purchased from Sigma-Aldrich (Beijing, China). Hexane and ethanol were purchased from Beijing Chemical Works (Beijing, China). All chemicals were directly used without any further purification.

### 2.2. Preparation of the Cs-Oleate Precursor

Cs_2_CO_3_ (0.2 g), 1 mL of OA, and 10 mL of ODE were stirred under vacuum for 50 min at 110 °C in a three-necked flask, and then the flask was heated to 150 °C under a constant Ar flow until the mixed solution became clear and transparent.

### 2.3. Synthesis of the CsPbBr_3_ NCs

Cubic CsPbBr_3_ NCs were synthesized following a well-established hot injection method. In total, 0.075 g of PbBr_2_, 0.7 mL of OLA, 5 mL of ODE, and 0.7 mL of OA were placed in the three-necked flask and dried under vacuum for 1 h at 110 °C. Then, the flask was maintained under a constant Ar flow for a further 30 min. After complete solubilization of the PbBr_2_ salt, the temperature was raised to 170 °C, and 0.2 mL of the Cs-oleate solution (prepared as described above) was quickly injected. Then, the reaction mixture was immediately quenched in an ice-water bath. Finally, the NCs were separated via centrifugation at 5000 rpm for 10 min.

### 2.4. Synthesis of the CsPbCl_x_Br_3−x_ NCs

PbBr_2_ and PbCl_2_ with different proportions, as well as 5 mL of ODE, 0.7 mL of OA, and 0.7 mL of OLA, were placed in a three-necked flask, with the details of the various experimental steps presented above. Finally, CsPbCl_x_Br_3−x_ (x = 1, 1.5, and 2) were successfully obtained.

### 2.5. Synthesis of the ZnCl_2_-Doped CsPbCl_1_Br_2_ NCs

The (ZnCl2)_x_-doped CsPbCl_1_Br_2_ (x = 5%, 10%, 20%) NCs were synthesized by adding ZnCl_2_. PbBr_2_, ZnCl_2_, and PbCl_2_ with different proportions, as well as 5 mL of ODE, 0.7 mL of OA, and 0.7 mL of OAM, were placed in a three-necked flask, with the details of the various experimental steps presented above. Finally, the NCs were separated via centrifugation at 5000 rpm for 10 min. The samples with 20% ZnCl_2_ are here denoted as ZnCl_2_-doped CsPbCl_1_Br_2_ perovskite NCs.

### 2.6. Post-Treatment

First, tetramethyl ammonium bromide with a concentration of 0.003 mol/L was used for treating the samples. The sample was stirred at room temperature for 10 min, and then it was allowed to stand for 20 min. Subsequently, specific amounts of ethyl acetate and didodecylammonium bromide were added. The sample was obtained after centrifugation.

### 2.7. Measurement and Characterization

The morphologies of the samples were characterized via transmission electron microcopy (TEM) and high-resolution TEM (HRTEM) using a JEM-2800 microscope (JEOL, Japan), operating at an accelerating voltage of 200 kV. The surface morphology of the samples was characterized using an SU-3500 scanning electron microscope (SEM) with an Oxford X-max 20 energy-dispersive X-ray spectrometer (EDS), manufactured by Hitachi, Tokyo, Japan. The crystalline structures of the as-obtained samples were analyzed via X-ray diffraction (XRD) using a Rigaku Smart-lab X-ray diffractometer (Rigaku, Tokyo, Japan) with Cu Kα radiation (λ = 1.5406 Å, current = 20 mA, and voltage = 40 kV). The PL emission spectra (both at room temperature and different temperatures) were acquired using a Techcomp FL970 fluorescence spectrometer (Techcomp, Beijing, China). The excitation source was a continuous-wave laser operating at a wavelength of 380 nm. For the time-resolved measurements, a pulsed laser with a pulse duration of 1 ns was used. The excitation intensity was set to 5 mW using a neutral density filter. The absorption spectra were recorded using a UV-Vis-NIR spectrophotometer (Shimadzu UV-3600iplus, Kyoto, Japan) with a spectral resolution of 1 nm in the wavelength range from 200 to 800 nm. The emission decay dynamics were monitored using the time-correlated single-photon counting capability of FLS1000s (1024 channels, 200 ns window), with 5000 data points being collected. The PLQY was measured using a fluorescence spectrometer (OmniFluo900, Beijing, China) with integrating sphere from Zolix. The system had a PLQY measurement accuracy of ±2%. The perovskite NCs were dispersed in hexane at a concentration of 0.05 mg/mL, and the measurements were performed on the solution in a quartz cuvette with a 1 cm path length. The temperature-dependent measurements were performed using a Zolix-LH101 (Beijing, China), which allowed temperature control in the range from 80 to 400 K with an accuracy of ±0.5 K. Temperatures were monitored using a silicon diode temperature sensor mounted near the sample. The temperature was calibrated against a standard reference prior to the measurements, ensuring an accuracy of ±0.5 K across the entire measurement range.

## 3. Results and Discussion

**Figure 1:**
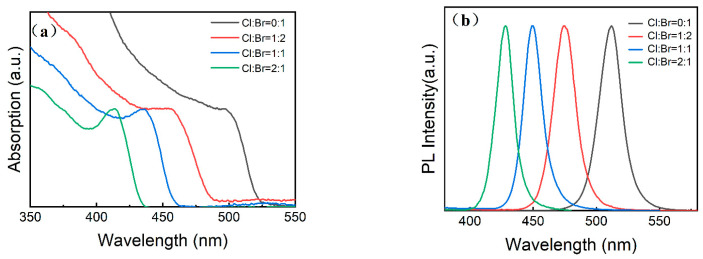
(**a**) UV-vis absorbance and (**b**) PL spectra of the initial CsPbBr_3_ and CsPbCl_x_Br_3−x_ (x = 1, 1.5, and 2) perovskite NCs.

The pure CsPbBr_3_ and CsPbCl_x_Br_3−x_ (x = 1, 1.5, and 2) perovskite NCs were prepared, and the Cl/Br ratio was modified by varying the PbBr_2_ and PbCl_2_ precursor feeding amount in the reaction medium (the details are provided in the Experimental Section). The UV-vis absorbance and normalized PL spectra of the initial CsPbBr_3_ and CsPbCl_x_Br_3−x_ (x = 1, 1.5, and 2) perovskite NCs are shown in Figure 1. These distinct absorption peaks corresponding to band-edge excitons in the CsPbBr_3_ and CsPbCl_x_Br_3−x_ (x = 1, 1.5, and 2) perovskite NCs can be observed. With the increase in chlorine content, the position of the emission peak is blue-shifted from 520 to 428 nm. As shown in Figure 1a, the optical band gap of the perovskite NCs, which increases upon increasing the chlorine amount, was determined from the relationship between the absorption coefficient (α) and the photon energy (*hv*) [30]. This increased bandgap is related to the smaller valence band maximum due to the replacement of the Br^−^ ions in the NCs with the Cl^−^ ions due to the addition of PbCl_2_. Compositional engineering of perovskite NCs is a typical approach to endow them with the capacity to emit light in the desired region, and the Br and Cl contents can be monotonically controlled to achieve deep-blue emission (460–480 nm) [31]. However, according to a previous report [32], the PLQY values of the CsPbCl_x_Br_3−x_ perovskite NCs obviously decrease with the increase in Cl^−^ content, indicating that this traditional method is not suitable for obtaining effective blue-emitting perovskite NCs. Thus, in this work, doping and post-treatment were performed on the prepared perovskite NCs.

**Figure 2:**
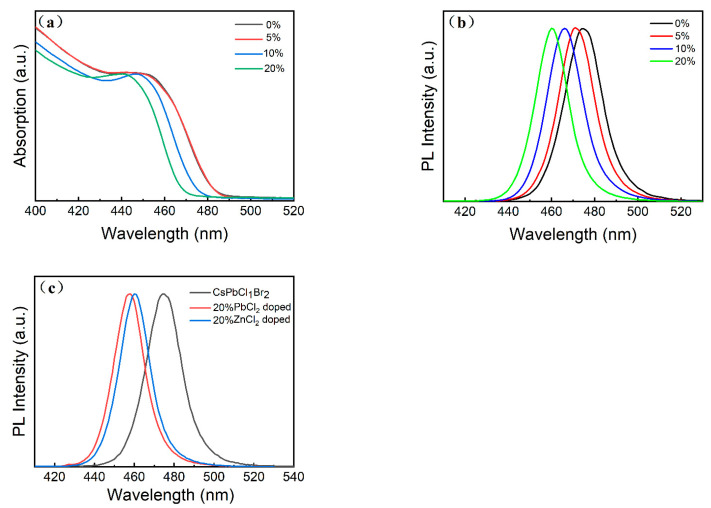
(**a**) UV-vis absorbance and (**b**) PL spectra of the undoped and ZnCl_2_-doped CsPbCl_1_Br_2_ perovskite NCs (5%, 10%, and 20%). (**c**) PL spectra of the CsPbCl_1_Br_2_, the ZnCl_2_-doped CsPbCl_1_Br_2_ (20%), and the PbCl_2_-doped CsPbCl_1_Br_2_ (20%) perovskite NCs.

To achieve pure-blue light emission (460–470 nm), the CsPbCl_1_Br_2_ perovskite NCs were doped with ZnCl_2_. In this work, a series of ZnCl_2_-doped CsPbCl_1_Br_2_ perovskite NCs were prepared via the hot injection method, in which different molar proportions of ZnCl_2_ (0%, 5%, 10%, and 20%) were incorporated into the original precursor. The UV-vis absorbance and PL spectra of the undoped and ZnCl_2_-doped CsPbCl_1_Br_2_ NCs (5%, 10%, and 20%) are shown in Figure 2. Distinct absorption peaks corresponding to band-edge excitons in the undoped and ZnCl_2_-doped CsPbCl_1_Br_2_ NCs (5%, 10%, and 20%) can be observed in Figure 2a. The PL spectra reveal that the CsPbCl_1_Br_2_ NCs exhibit a PL peak at 475 nm, while the ZnCl_2_-doped CsPbCl_1_Br_2_NCs display a PL peak at 460 nm (Figure 2b) with an FWHM of less than 20 nm. The PL peaks are blue-shifted as the ZnCl_2_ doping amount increases. Similarly, the exciton peak in the absorption spectra also shifts to a larger optical band gap. Once ZnCl_2_ is added to the precursor solution, the PL peak of the obtained ZnCl_2_-doped CsPbCl_1_Br_2_ NCs is substantially blue-shifted. The blue-shifting of the PL wavelength becomes more pronounced with increasing Cl ion content [33]. This increased bandgap is related to the reduction in valence band maximum due to the substitution of the Br^−^ ions in the NCs with the Cl^−^ ions [10]. The incorporation of metal ions into lead halide perovskite NCs often results in a blue shift in the absorption peak and the PL peak when the metal ions are smaller than the Pb^2+^ ions [34]. In a previous work [35], they fabricated zinc-doped perovskite NCs via the post-treatment method. This involved adding zinc bromide salt to a toluene solution of pre-synthesized CsPbBr_3_ perovskite NCs. Notably, no substantial alteration in the size of the NCs was observed. Thus, the blue shift observed in our study can be attributed to ZnCl_2_ doping. Huang et al. reported that highly luminescent zinc-doped CsPbBr_3_ perovskite NCs exhibited a distinct blue-shifted emission peak precisely at 503.7 nm, accompanied by a notably narrower spectral width (18.7 nm), showcasing unique optical characteristics [36]. No additional peak related to the dopant-induced defect levels can be observed, unlike in the case of Mn^2+^-, Yb^3+^-, and Ce^3+^-doping [34,37]. To further investigate the influence of the zinc ions on the PL emission peak, we prepared 20% PbCl_2_-doped CsPbCl_1_Br_2_ NC samples. It is found that the emission peak of the sample is significantly blue-shifted to 458 nm, as shown in Figure 2c, indicating that the introduction of the zinc ions hinders the entrance of lead and chloride ions into the lattice.

**Figure 3:**
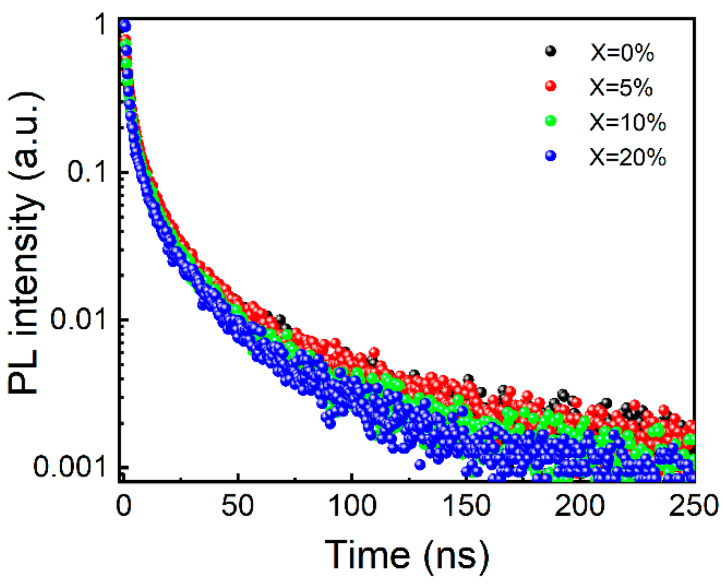
Decay curves of time-resolved PL of the undoped and ZnCl_2_-doped CsPbCl_1_Br_2_ perovskite NCs (5%, 10%, and 20%). The curves were fitted with a stretched-exponential function. λexc = 450 nm; τpulse = 1 ns; f = 10 kHz; I exc = 5 mW/cm^2^.

The defects present in a semiconductor are crucial in determining its performance. For instance, the lifetime of perovskite NCs can be significantly influenced by the presence of defects. Multiexponential decay in CsPbX_3_ (where X = Br, I and Cl) NCs, with lifetimes ranging from approximately 1 to 30 ns, has been reported in the literature [38]. The multiexponential decay in CsPbX_3_ NCs is likely associated with their intrinsic defects, such as vacancies, which have a relatively low formation energy [39]. Time-resolved PL spectra (Figure 3) were collected to gain deeper insights into the exciton recombination dynamics. As shown in Figure 3, the PL decay curve can be well fitted using the triexponential function:

$$A\left(t\right)=A_{1}\;\exp\left(-\frac{t}{\tau_{1}}\right)+A_{2}\;\exp\left(-\frac{t}{\tau_{2}}\right)+A_{3}\;\exp\left(-\frac{t}{\tau_{3}}\right)$$

where *A*_1_, *A*_2_, and *A*_3_ are constants, and *t* is time. The average lifetime (τ_avg_) can be calculated as follows:

$$\tau_{avg}=\frac{A_{1}\tau_{1}^{2}+A_{2}\tau_{2}^{2}+A_{3}\tau_{3}^{2}}{A_{1}\tau_{1}+A_{2}\tau_{2}+A_{3}\tau_{3}}$$

The obtained samples exhibit three lifetimes (*τ*), shown in Table 1, which reveals the high proportion of radiative recombination to nonradiative transitions. It is observed that the carrier lifetime of the ZnCl_2_-doped CsPbCl_1_Br_2_ NCs is shorter than that of CsPbCl_1_Br_2_ NCs (12.02 ns). Abnormally, the τ_avg_ of ZnCl_2_-doped CsPbCl_1_Br_2_ NCs is shorter than that of CsPbCl_1_Br_2_ NCs. In addition to the intrinsic defects, the triexponential decay and the shorter lifetimes of the ZnCl_2_-doped CsPbCl_1_Br_2_ Ncs can be ascribed to the increased number of vacancies with increasing Cl^−^ ion content. Bromine and chlorine ion vacancies (V_Br_ and V_Cl_) are easily formed within lead halide perovskite NCs due to their extremely low formation energy [40]; these vacancies can swiftly capture excitons and cause the rapid attenuation of the optical performance of the NCs. Hence, the fact that the surface defects have been almost completely eliminated is the critical reason for the observed enhancement of the optical properties of the perovskite NCs. In ZnCl_2_-doped CsPbCl_1_Br_2_ NCs, the formation of Br and Cl vacancies is very common. Cl vacancy defects, whose concentration increases with increasing ZnCl_2_ doping amount, behave as luminescence quenchers. The excitons are quickly captured by the Cl vacancy defects. This decreases the band-edge exciton density and promotes nonradiative recombination. These findings are in agreement with the results reported by Jiang [32]. Additionally, it can also be seen that Zn^2+^ doping can effectively reduce the nonradiative recombination caused by vacancy defects. Generally, a low PLQY is mainly caused by the presence of halide vacancy defects on the NC surface [41]. For the ZnCl_2_-doped CsPbCl_1_Br_2_ NCs, the PLQY decreases from 85% to 73%, as shown in Table 1. Upon doping with zinc chloride, an increase in the zinc doping content leads to a concomitant rise in chlorine content. The PLQY is governed by the competition between zinc ions and chloride ions. The decline in PLQY subsequent to doping is primarily ascribed to the dominant role played by the nonradiative recombination processes induced by chlorine vacancy defects. On the other hand, for the PbCl_2_-doped CsPbCl_1_Br_2_ NCs, the PLQY is only 47%. Therefore, it can be concluded that the reason why Zn^2+^ doping can improve the PLQY of CsPbCl_1_Br_2_ NCs is that its incorporation can greatly increase the radiation decay rate and at the same time inhibit the nonradiative decay rate.

**Figure 4:**
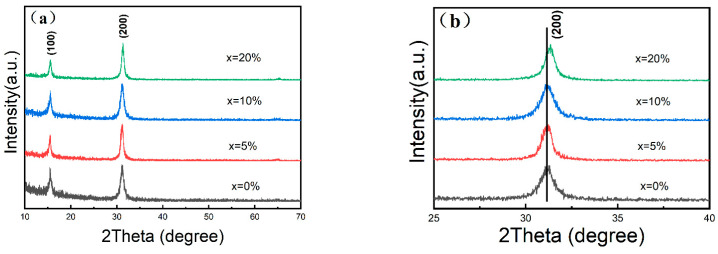
(**a**) XRD patterns of the undoped and ZnCl_2_-doped CsPbCl_1_Br_2_ perovskite NCs (5%, 10%, 20%) and (**b**) enlarged XRD patterns in peak position of (200) lattice plane.

To study the structure of the samples, XRD measurements were carried out. As the XRD patterns in Figure 4 show, the undoped and ZnCl_2_-doped CsPbCl_1_Br_2_ NCs(5%, 10%, and 20%) have similar diffraction peaks, indicating that perovskite phases (Portable Document Format (PDF) #54-0752) are formed in both the undoped CsPbCl_1_Br_2_ and ZnCl_2_-doped CsPbCl_1_Br_2_ NCs with different Zn/Pb molar ratios. It can be clearly observed that the introduction of the Zn^2+^ ions does not significantly change the chemical structure of the obtained samples nor does it generate impurities in the system. The (200) diffraction peaks shift to a higher angle as the Zn^2+^ doping amount increases, confirming that the lattice is slightly contracted (Figure 4b). This can be explained by the fact that the radius of the Pb^2+^ ions (119 pm) is larger than that of the Zn^2+^ ions (74 pm) [3]. According to this result and a previous work [42], it is concluded that zinc ions have been successfully incorporated into the perovskite lattice.

**Figure 5:**
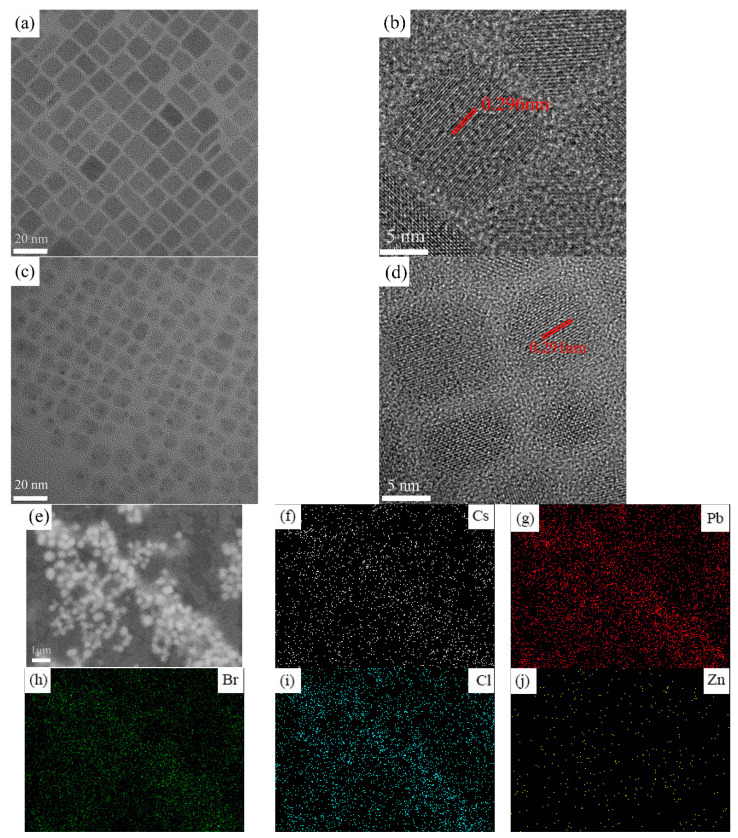
TEM images of (**a**) the undoped and (**b**) the ZnCl_2_-doped CsPbCl_1_Br_2_ perovskite NCs (20%). HRTEM images of (**c**) the undoped and (**d**) the ZnCl_2_-doped CsPbCl_1_Br_2_ perovskite NCs (20%). (**e**) SEM image of the ZnCl_2_-doped CsPbCl_1_Br_2_ perovskite NCs(20%). (**f**–**j**) Elemental mapping of Cs, Pb, Cl, Br, and Zn in the ZnCl_2_-doped CsPbCl_1_Br_2_ perovskite NCs.

TEM and HR-TEM imaging of the undoped CsPbCl_1_Br_2_ and the ZnCl_2_-doped CsPbCl_1_Br_2_ perovskite NCs were carried out, and the corresponding results are shown in Figure 5a–d. All the perovskite NCs show uniform cubic crystals. Additionally, for the CsPbCl_1_Br_2_ perovskite NCs, the average NC size is 9.6 nm, as shown in Figure 5a. However, for the ZnCl_2_-doped CsPbCl_1_Br_2_ perovskite NCs, the average NC size is lower (8.7 nm), as shown in Figure 5c. As can be seen from Figure 5c, there are several small black dots in the image, which originate from the Pb^0^ particles formed by the structural dissociation of the perovskite NCs with a soft lattice structure under electron beam irradiation [43]. This may be due to the introduction of the zinc ions, which would result in an excess of lead ions. From the HR-TEM images, it can be found that the (200) lattice spacing decreases from 0.296 nm (Figure 5b) to 0.291 nm (Figure 5d). These results are in good agreement with the XRD findings (Figure 4), which further demonstrates that the Zn^2+^ ions were successfully incorporated into the CsPbCl_1_Br_2_ perovskite lattice. Due to the replacement of Pb^2+^ with Zn^2+^, which has a smaller ionic radius, the resulting lattice contraction of the CsPbCl_1_Br_2_ NCs gives rise to a smaller plane spacing. The effect of Zn^2+^ doping on the morphology of the CsPbCl_x_Br_3−x_ perovskite NCs is thus mainly a change in grain size. This may result from the fact that zinc ions attract charges more strongly than Pb^2+^ ions; thus, Zn^2+^ ions are both easier to attract and be attracted by the surrounding Br^−^ ions during the formation of the CsPbCl_1_Br_2_ perovskite NCs than the Pb^2+^ ions [44]. Furthermore, from the EDS mappings presented in Figure 5e–j, it is seen that all the elements in the ZnCl_2_-doped CsPbCl_1_Br_2_ perovskite NCs are homogenously distributed, and zinc ions are evenly distributed in the perovskite NCs. From both the XRD and TEM results, it can be safely concluded that the Zn ions have been successfully incorporated into the CsPbCl_1_Br_2_ perovskite NC lattice, and have occupied a fraction of the Pb^2+^ sites rather than the Cs^+^ and Br^−^ sites.

**Figure 6:**
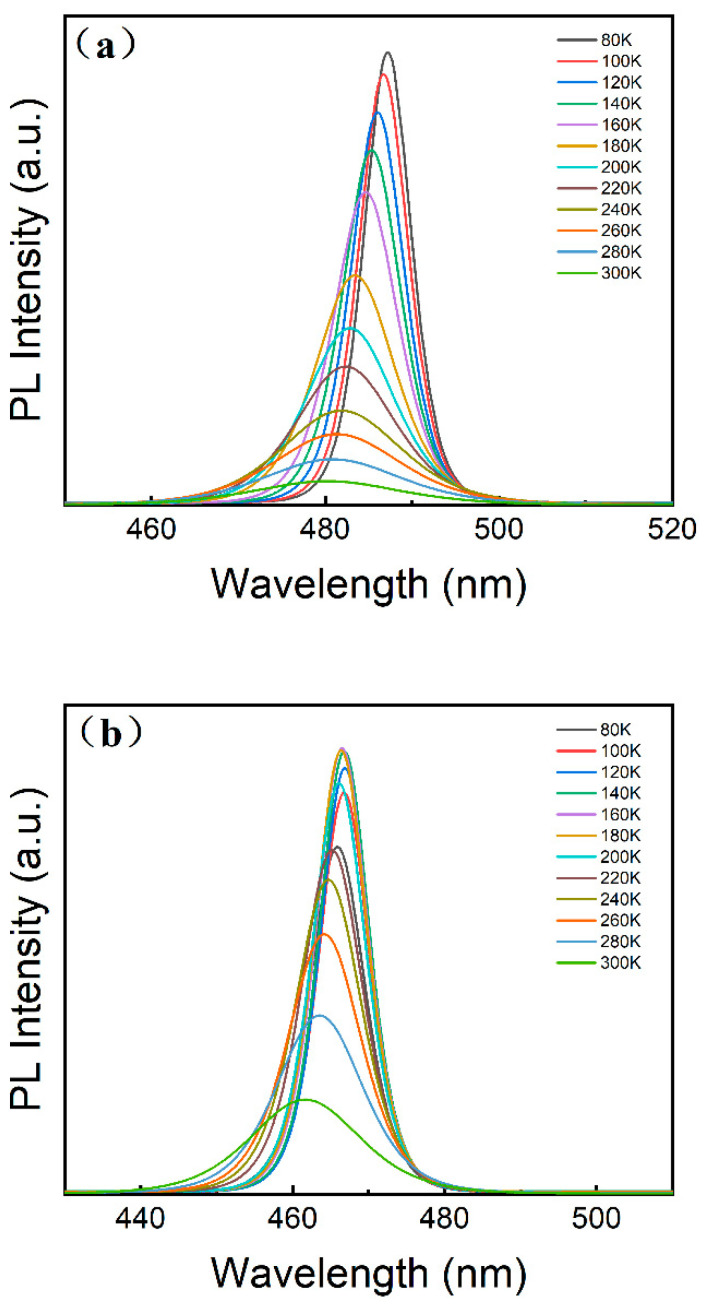
PL spectral evolution of (**a**) the undoped and (**b**) ZnCl_2_-doped CsPbCl_1_Br_2_ (20%) perovskite NCs at different measurement temperatures.

To further explore the PL decay mechanism in the CsPbCl_1_Br_2_ and ZnCl_2_-doped CsPbCl_1_Br_2_ perovskite NCs, temperature-dependent PL spectra were acquired in the temperature range of 80–300 K. For the CsPbCl_1_Br_2_ perovskite NCs (Figure 6a), the PL intensity decreases progressively as the temperature increases from 80 to 300 K, indicating enhanced trapping of the charge carriers at higher temperatures, which is due to the increasing number of nonradiative recombination centers. However, the PL intensity of the ZnCl_2_-doped CsPbCl_1_Br_2_ perovskite NCs increases when the temperature increases from 80 to 200 K (Figure 6b). Then, the PL intensity decreases when the temperature increases from 200 to 300 K, a behavior that is referred to as negative thermal quenching. The observed negative thermal quenching phenomenon can be accounted for by the dynamic competition between shallow trap states and excitonic states. When zinc ions are incorporated into the perovskite NCs, due to the substantial difference in ionic radius between Zn^2+^ (0.074 nm) and Pb^2+^ (0.119 nm), shallow trap states are introduced into the crystal lattice. In the temperature range from 80 to 200 K, charge carriers (either electrons or holes) can easily be trapped by these shallow traps. As the thermal energy kT approaches the activation energy of the shallow traps, the trapped carriers can escape the traps and re-engage in radiative recombination. At this moment, the excitonic states are strengthened as a result of carrier replenishment, and the PL intensity increases with the rise in temperature, thereby giving rise to negative thermal quenching. When the temperature rises from 200 to 300 K, thermal activation (with an increase in kT) enables the carriers to escape from the traps and return to either the conduction band or the excitonic states. However, nonradiative recombination then dominates, leading to a decline in the PL intensity. This intriguing PL behavior can be elucidated by considering the participation of the trap states situated in close proximity to the excitonic states during the trapping–detrapping process. At specific temperatures, the charge carriers trapped within these shallow trap states can return to the excitonic states and subsequently undergo radiative recombination. However, at elevated temperatures, the insufficient thermal energy (*kT*) may prevent the effective repopulation of the excitonic state with carriers. This is why a drop in PL intensity is observed. This may cause an improvement of the thermal antiquenching performance, implying better PL thermal stability. This will provide new insights into the design of high-efficiency, stable light-emitting and detection devices.

**Figure 7:**
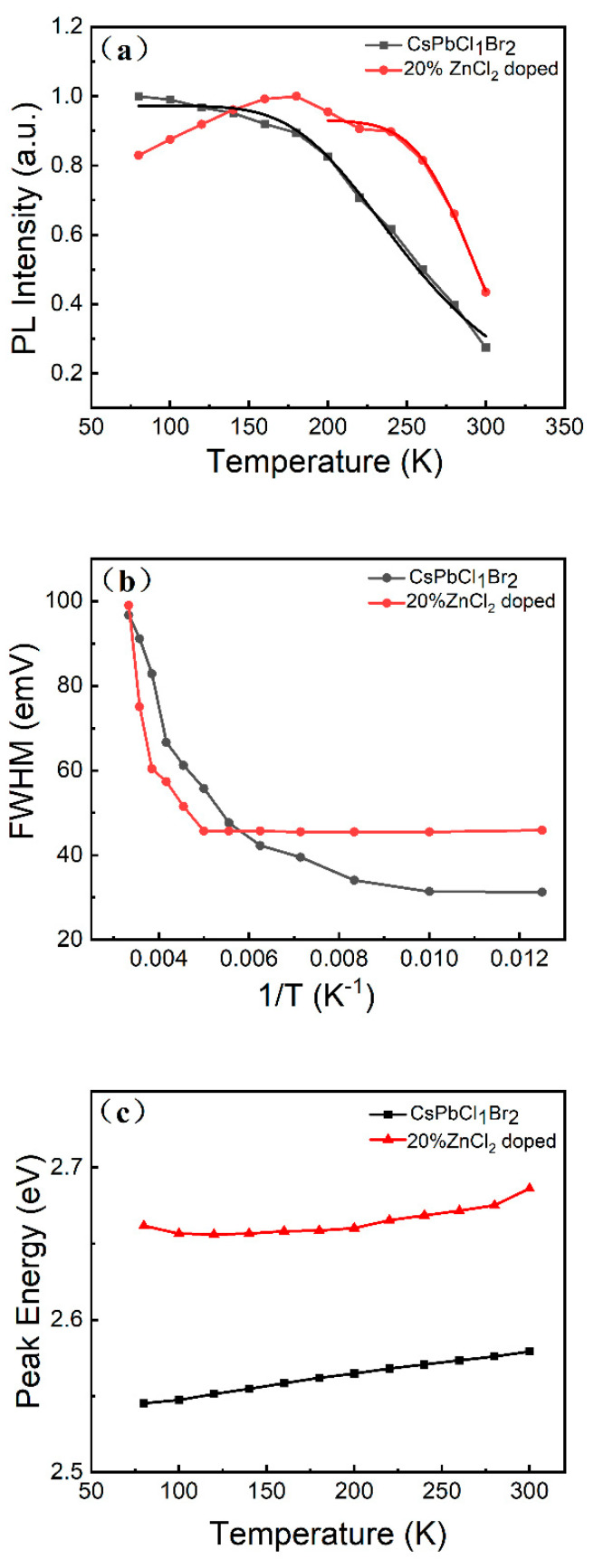
(**a**) Integrated PL intensity, (**b**) FWHM values, and (**c**) peak energies as a function of temperature of the undoped and ZnCl_2_-doped CsPbCl_1_Br_2_ (20%) perovskite NCs.

To extract the exciton binding energy, the integrated PL emission intensity (Figure 7a) is plotted as a function of temperature using the Arrhenius equation [45], that is

$$I\left(T\right)=\frac{I_{0}}{1+Aexp\left(-E_{b}/k_{B}T\right)}$$

where *I*_0_, *E_b_*, and *k_B_* are the intensity at 0 K, the exciton binding energy, and the Boltzmann constant, respectively. From the fitting, it can be seen that the CsPbCl_1_Br_2_ perovskite NCs have a large exciton binding energy (128 meV), while the ZnCl_2_-doped CsPbCl_1_Br_2_ perovskite NCs exhibit an *E_b_* of 354 meV (Figure 7a). Importantly, a larger exciton binding energy is indicative of a higher PL intensity [46].

In addition, for the CsPbCl_1_Br_2_ perovskite NCs, as the temperature increases, the FWHM also gradually increases. The exciton–optical phonon interaction plays a dominant role in PL linewidth broadening. The exciton–phonon interaction occurring during the carrier recombination process was analyzed in terms of the variation in FWHM with temperature according to the following equation:

$$\Gamma \left(T\right)=\Gamma_{0}\sigma T+\frac{\Gamma_{op}}{\exp\left(\hbar \varpi_{op}/KT\right)-1}$$

where Γ_0_ is the inhomogeneous broadening contribution, *σ* and *Γ_op_* are the exciton–acoustic phonon and exciton–optical phonon interaction contributions to the linewidth broadening, respectively, $\hbar \varpi_{op}$ represents the optical phonon energy, and Γ(*T*) is the FWHM value at the measured temperature *T* [47]. As shown in Figure 7b, for the CsPbCl_1_Br_2_ perovskite NCs, the FWHM increases steadily as the temperature ranges from 80 to 300 K. However, for the ZnCl_2_-doped CsPbCl_1_Br_2_ perovskite NCs, the FWHM remains unchanged with the temperature ranging from 80 to 200 K, and then the FWHM increases at a faster rate with temperature continuing to increase from 200 to 300 K, suggesting the presence of strong exciton–phonon interactions during the excitons’ recombination process. This phenomenon gives rise to a non-linear increase in FWHM with temperature. This may result in an improvement in the thermal antiquenching performance of the NCs, implying better PL thermal stability.

Furthermore, for the CsPbCl_1_Br_2_ perovskite NCs, as shown in Figure 7c, the PL peak shows a clear blue shift in this temperature range, from 487 to 480 nm. This maybe due to the electro–phonon coupling of the NCs, and a similar temperature-dependent emission behavior was also observed in the CsPbBr_3_ perovskite NCs [48]. However, for the ZnCl_2_-doped CsPbCl_1_Br_2_ perovskite NCs (Figure 7c), the PL peak does not shift as the temperature increases from 80 to 200 K. Only as the temperature continues to increase to 300 K does the PL peak show a significant blue shift from 466 to 460 nm.

**Figure 8:**
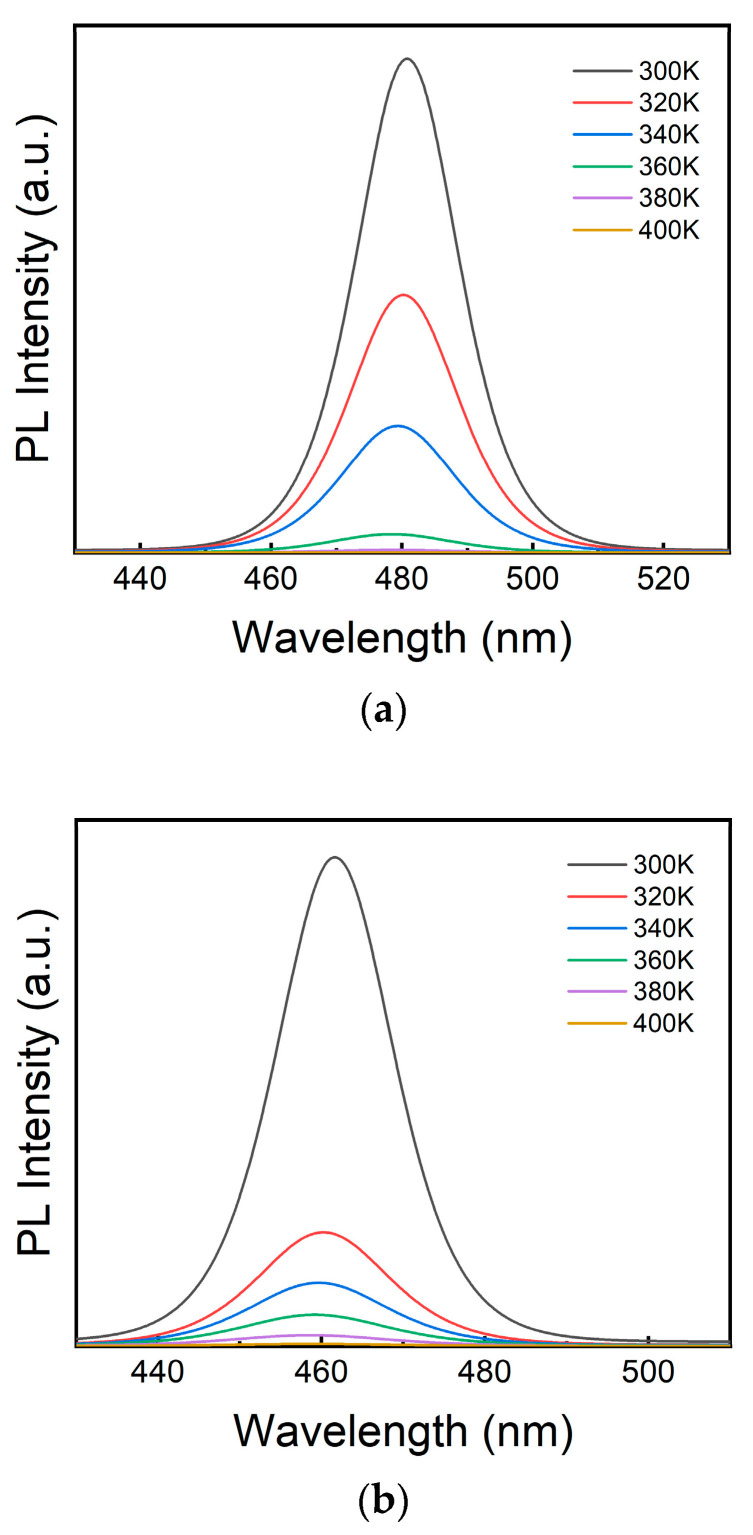
PL spectral evolution of (**a**) the undoped and (**b**) ZnCl_2_-doped CsPbCl_1_Br_2_ (20%) perovskite NCs at temperatures ranging from 300 to 400 K.

To investigate the high-temperature stability of the perovskite NCs, temperature-dependent PL spectra were acquired from 300 to 400 K for both the undoped and ZnCl_2_-doped CsPbCl_1_Br_2_ perovskite NCs, as shown in Figure 8a,b. In the temperature range from 300 to 400 K, the intensity of PL emission peak of the ZnCl_2_-doped CsPbCl_1_Br_2_ perovskite NCs decreases more rapidly after doping with zinc ions, which is essentially closely related to the temperature-induced enhancement of the nonradiative recombination and the modulation of the perovskite structure/defect states achieved through doping. In general, the PL intensity of perovskites is determined by the competition between radiative recombination and nonradiative recombination. Nonradiative recombination usually dominates when the temperature rises (because the thermal motion of carriers is enhanced, making it easier for them to dissipate energy through interaction with defects, phonons, and other paths). In the process of Zn^2+^ doping, the content of Cl^−^ also increases. In this case, the defects introduced by Cl^−^ play a dominant role at high temperatures, promoting nonradiative recombination and leading to an accelerated decrease in the intensity of the PL peak. So at high temperatures (300–400 K), Cl^−^ ions play a dominant role in the changes in the PL emission peak intensity of perovskite NCs.

**Figure 9:**
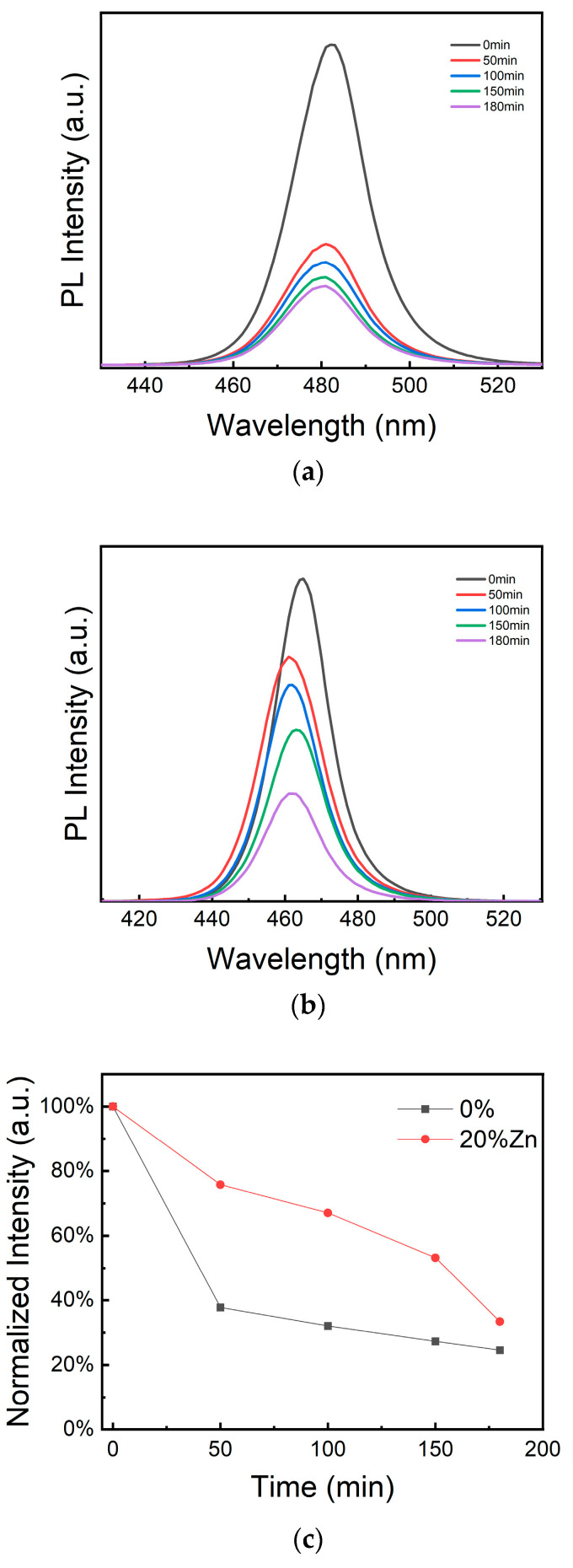
PL spectral evolution of (**a**) the undoped and (**b**) ZnCl_2_-doped CsPbCl_1_Br_2_ (20%) perovskite NCs. (**c**) PL peak intensity under different ultraviolet irradiation time.

To further assess the stability of the perovskite NCs, ultraviolet (UV)-lamp irradiation tests were performed. Figure 9a,b present the PL spectra of the undoped and ZnCl_2_-doped CsPbCl_1_Br_2_ perovskite NCs, respectively, under UV irradiation for over 200 min. As illustrated in Figure 9c, the intensity of the PL peak for the ZnCl_2_-doped CsPbCl_1_Br_2_ perovskite NCs remained higher than that of the undoped counterpart in each time interval. These results confirm that Zn doping effectively enhances the stability of the perovskite NCs.

**Figure 10:**
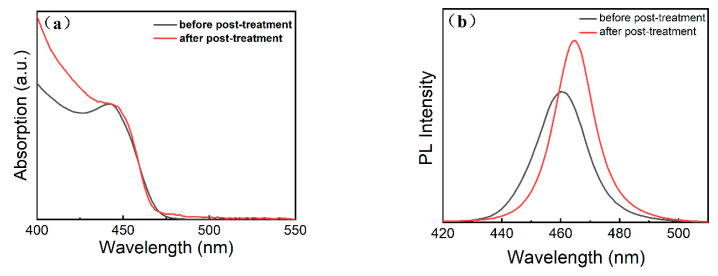
(**a**) UV-vis absorption and (**b**) PL spectra of the ZnCl_2_-doped CsPbCl_1_Br_2_ (20%) before and after post-treatment.

To improve the PLQY, the ZnCl_2_-doped CsPbCl_1_Br_2_ perovskite NCs were post-treated. The UV-vis absorbance and normalized PL spectra of the original ZnCl_2_-doped CsPbCl_1_Br_2_ perovskite NCs and the post-treated NCs are shown in Figure 10. The PL peaks show an obvious red shift after the post-treatment, with their intensity increasing significantly. The PLQY of the post-treated samples reaches the maximum value of 83%. This can be attributed to the gradual substitution of the Cl^−^ ions by the Br^−^ ions [32]. This demonstrates the efficiency and feasibility of the post-treatment strategy for synthesizing high-fluorescence blue-emissive perovskite NCs. The elimination of a substantial number of surface defects is the critical reason for the observed enhancement of the optical properties of the post-treated perovskite NCs.

## 4. Conclusions

ZnCl_2_-doped CsPbCl_1_Br_2_ perovskite NCs were fabricated through a facile hot injection method using ZnCl_2_ as the Zn source. Not only can the emission wavelength of ZnCl_2_-doped CsPbCl_1_Br_2_ perovskite NCs be easily adjusted from 475 to 460 nm, but also the PLQY of the NCs is greatly improved due to the efficient removal of the surface defects in the NCs. A blue emission at 460 nm with a PLQY of 73% is achieved for the ZnCl_2_-doped CsPbCl_1_Br_2_ perovskite NCs, while the PLQY is only 42% for the PbCl_2_-doped CsPbCl_1_Br_2_ perovskite NCs. Furthermore, after the post-treatment, the PLQY increases significantly from 73% to 83%. Finally, zinc doping is beneficial for enhancing the stability of the perovskite NCs. These findings provide new insights for investigating other Zn^2+^-doped perovskite materials.

## Figures and Tables

**Table 1:** Fitting parameters of PL decay curves of the undoped and ZnCl_2_-doped CsPbCl_1_Br_2_ perovskite NCs (5%, 10%, and 20%) fitted by triexponential function and PLQY.

Sample	A_1_ [%]	τ_1_ [ns]	A_2_ [%]	τ_2_ [ns]	A_3_ [%]	τ_3_ [ns]	τ_ave_ [ns]	PLQY
X = 0%	1.21	0.87	0.34	4.84	0.06	26.71	12.02	85%
X = 5%	1.09	0.8	0.41	4.03	0.09	21.67	11.09	81%
X = 10%	1.21	0.79	0.32	4.35	0.08	21.56	10.80	78%
X = 20%	2.1	0.8	0.26	4.96	0.06	24.76	9.99	73%

## Data Availability

The data that support the findings of this study are available from the corresponding authors, J.H. and X.Y., upon reasonable request.
